# ADAMTS13 in the New Era of TTP

**DOI:** 10.3390/ijms25158137

**Published:** 2024-07-26

**Authors:** Anna Papakonstantinou, Panagiotis Kalmoukos, Aikaterini Mpalaska, Evaggelia-Evdoxia Koravou, Eleni Gavriilaki

**Affiliations:** 1 Faculty of Health Sciences, School of Medicine, Aristotle University of Thessaloniki, 54124 Thessaloniki, Greece; 2 2nd Propedeutic Department of Internal Medicine, Aristotle University of Thessaloniki, 54642 Thessaloniki, Greece; kalmoukosp@yahoo.gr (P.K.); aikatbal@yahoo.gr (A.M.); evkoravou@gmail.com (E.-E.K.)

**Keywords:** ADAMTS13 cleaving protease, thrombotic thrombocytopenic purpura, plasmic score, rituximab, caplacizumab, recombinant ADAMTS13

## Abstract

Thrombotic thrombocytopenic purpura (TTP) is a life-threatening, often immune-mediated disease that affects 2–13 persons per million per year. Hemolytic anemia, thrombocytopenia, and end-organ damage due to the formation of microthrombi are characteristic of TTP. ADAMTS13 is a disintegrin, metalloproteinase, cleaving protein of von Willebrand factor (VWF) that processes the VWF multimers to prevent them from interacting with platelets and, in turn, to microvascular thrombosis. Prompt diagnosis of TTP is critical yet challenging. Thrombotic microangiopathies have similar clinical presentation. Measurement of ADAMTS13 activity helps in the differential diagnosis. Less than 10% ADAMTS13 activity is indicative of TTP. Laboratory ADAMTS13 activity assays include incubating the test plasma with the substrate (full-length VWM multimers) and detection with direct or indirect measurement of the cleavage product. The purpose of this study is to examine the diagnostic potential, advantages, and weaknesses of the ADAMTS13 potency in TTP.

## 1. Introduction

### 1.1. ADAMTS13 Activity as a Diagnostic Means to TTP

Thrombotic thrombocytopenic purpura (TTP) is a life-threatening, often immune-mediated type of thrombotic microangiopathy (TMA) that affects 2–13 persons per million per year. Thrombotic microangiopathies are characterized by a clinical triad of hemolytic anemia, thrombocytopenia, and end-organ damage due to microvascular thrombosis [1]. The study aims to examine the diagnostic potential, advantages, and weaknesses of the ADAMTS13 in thrombotic thrombocytopenic purpura (TTP) and present novel approaches to the diagnosis, treatment, and follow-up of TTP patients, which assimilates both clinical and laboratory data.

TTP is distinguishable from other TMAs due to different pathophysiology and treatment options, and mainly due to patients’ severe deficiency of ADAMTS13, a metalloproteinase, disintegrin, with a thrombospondin type 1 motif, member 13, which processes the von Willebrand factor (VWF) multimers to prevent them from interacting with platelets and form microvascular thrombi [2,3]. ADAMTS13, found in the plasma under physiological conditions, circulates in “closed” conformation. In contrast, it is found in open conformation during the acute episode of iTTP and in clinical remission with reduced ADAMTS13 levels [4]. The study by South et al. showed that the epitope in the cryptic spacer domain is the core antigenic target for anti-ADAMTS13 inhibitors in iTTP [5]. In TTP patients, the targets of the polyclonal antibodies are various ADAMTS13 domains, but the spacer domain is the preferred attachment of the inhibitory antibodies. Recent evidence suggests that the antibodies to the distal C-terminal domains of the ADAMTS13 activate it by removing its inhibition. It also suggests that the anti-C-terminal antibodies change the ability of the inhibitory antibodies towards ADAMTS13 activity [6,7].

Patients with congenital TTP (cTTP) have severe ADAMTS13 deficiency due to biallelic mutations in the coding gene—Upshaw–Shulman syndrome, an autosomal recessive disease. The French Registry study showed that *ADAMTS13* gene mutations impact the ADAMTS13 conformation and the disease’s age onset in patients with congenital TTP [8]. On the other hand, patients with immune-mediated TTP (iTTP) have anti-ADAMTS13 inhibitors that counteract the ADAMTS13 function, leaving VWF polymers uncleaved. TTP is associated with the pregnancy state in 10–30% of all adult TTP patients [9] and has a clear relation with inherited mutations in the *ADAMTS13* gene (adult-onset cTTP) [10].

While the ADAMTS13 activity levels have a clear correlation with the clinical diagnosis of TTP, the role of quantitative measurement of ADAMTS13 antigen (ADAMTS13:Ag) is poorly understood yet evaluated in the clinical context of TTP. Recent studies have addressed a component of ADAMTS13:Ag in TTP prognosis during treatment and follow-up [11], linking five-fold higher mortality with ADAMTS13:Ag in the lowest quartile at diagnosis [12]. It is thought-provoking that TTP patients with low ADAMTS13 activity have varied levels of ADAMTS13:Ag, implying independence between detected ADAMTS13:Ag levels and ADAMTS13 enzyme activity [12]. Detected ADAMTS13:Ag levels presumably include free protein, protein bound to antibody inhibitors (in immune TTP), and in complex with other carriers. Thus, ADAMTS13 deficiency is not necessarily linked to low ADAMTS13:Ag levels in TTP patients [13].

ADAMTS13 inhibition has been a topic of interest in recent years. iTTP patients have ADAMTS13 deficiency in common, but not all have inhibitory autoantibodies [14]. Studies show that iTTP patients (both in acute and remission phases) produce autoantibodies that inhibit ADAMTS13 activity in processing VWF multimers. These inhibitors are directed against the spacer domain in three different hotspot regions of the ADAMTS13 [15]. The inhibitors have 16 distinct antispacer epitope profiles. Bethesda-like assays detect the presence of these functional inhibitors [16]. However, non-functional inhibitory antibodies may also be present, boosting the clearance of ADAMTS13 from circulation, and are detected by assays like ELISA [16]. It must be noted that detected IgG autoantibodies against ADAMTS13 may be confused from the assays with other non-ADAMTS13 antibodies present in patients suffering from another autoimmune condition. Naturally, discrepancies between different methods of ADAMTS13 inhibitors exist [17]. Finally, a recent study by Saito et al. showed that the persistence of ADAMTS13 inhibitors postpones ADAMTS13 activity normalization in caplacizumab-treated iTTP patients, underlining the autoimmune character of this disease [18].

Despite their rarity, thrombotic microangiopathies have overlapping clinical presentations, from non-specific symptoms to major neurological episodes. Complement-mediated hemolytic uremic syndrome (CM-HUS) has served as a model disease for TMAs [19]. Understanding their pathophysiology is the key to distinguishing TTP from complement-mediated hemolytic uremic syndrome (CM-HUS) and other TMAs. Prompt diagnosis of TTP is critical yet challenging, as therapeutic plasma exchange (PEX) in the acute setting may be life-saving [20]. Measurement of ADAMTS13 activity helps in the differential diagnosis of TMAs, as ADAMTS13 activity of less than 10% indicates TTP [2]. 

Recent studies assessed the relationship between complement activation and TTP [21,22], showing that the alternative complement pathway may implicate in endothelial cells, neutrophils, and platelets’ activation [23], leading to the presence of ultra-large VWF multimers and microthrombi formation and consecutive hemolytic anemia. Preclinical studies showed that congenital ADAMTS13 deficiency and homozygous complement factor H deficiency produce severe thrombotic microangiopathy in mice [24]. Finally, the study by Cugno et al. showed that the highest activation levels of the complement system are observed in the acute phase of TTP, specifically in patients with renal impairment [23]. This relationship between renal alterations and high complement activation underlines the complement’s role in end-organ (kidney) damage [25] and thus shows the way for further studies to explore the use of complement inhibition in TTP patients.

Laboratory ADAMTS13 activity assays include incubating the test plasma with the substrate (full-length VWM multimers) and detection, with direct (quantification of the cleavage product) or indirect (quantification of the residual von Willebrand factor) measurement, which corresponds to the ADAMTS13 activity in the plasma. Fluorescence resonance energy transfer (FRET), gel electrophoresis, Western blotting, mass spectrometry, and chromogenic approaches are assays of direct measurement. In contrast, collagen-binding assays, enzyme-linked immunosorbent assay (ELISA), and ristocetin-induced aggregation are indirect assays of residual vWF determination. The measured value is presented as the percentage of normal pooled plasma after calibration and definition as 100% activity. The most frequently used methods are FRETS-VWF73 assay and collagen-binding assays [1]. However, the FRETS-VWF73 assay, using a 73-amino-acid peptide as substrate, is validated and considered better than collagen-binding assays [26].

These assays, nonetheless, have limitations. For example, FRETS-based assays are hampered with hyperbilirubinemia [27]. At the same time, plasma proteases, free hemoglobin, high endogenous VWF, and hyperlipidemia may also intervene with ADAMTS13 measurement in vitro, producing a misguiding low activity level in most assays [28,29]. In all circumstances, plasma should be drawn for ADAMTS13 activity measurement before initiation of therapeutic plasma exchange. It must be noted that results’ variations exist among different types of assays [16,17], which can be explained by the variety of variables in assay methodology and samples’ quality. Clinicians should be aware of their institutions’ methods’ availability and their limitations. They should request a second assay with a different methodology in cases where the ADAMTS13 activity results do not correspond with the patient’s clinical situation.

However, the timely availability of the results is only sometimes the case when guiding early treatment decisions [17]. It is, therefore, of utmost importance to identify more accurately patients with TTP who will benefit from PEX based on readily available information. ADAMTS13 activity assays include enzyme-linked immuno-absorbent assay (ELISA), fluorescence resonance energy transfer (FRETS)-VWF73, AcuStar, and Ceveron FRET. A recent study evaluated the sensitivity, specificity, and other qualities of these assays in accurately and promptly diagnosing TTP. (Table 1) [30].

### 1.2. TTP Disease Course

In their fourth decade, adult women often present with iTTP, with a female-to-male ratio of 2–3:1. iTTP seems to also favor the black race compared to non-blacks. Infants and children presenting with TTP are regarded as having the congenital form of TTP [33]. However, a recent French study showed that mutations of the *ADAMTS13* gene were present in approximately 3% of adult-onset TTP patients in occurrence with their first pregnancy [8]. Thus, pregnant women who present with a first episode of TTP should be assessed for the inherited form of the disease. 

iTTP may be isolated (primary) or associated with another underlying condition (secondary), such as autoimmune diseases, cancer, infection, pregnancy, or drug-induced. Predisposing conditions are established in 27–69% of patients with severe ADAMTS13 deficiency [8,34,35]. The primary diagnostic algorithm of a focused history, thorough clinical examination, and laboratory data should be applied to all TTP patients to exclude a secondary iTTP. A recent study from the United Kingdom TTP Registry showed that neurological manifestations were more frequent and more profound in primary TTP and that most patients presented with stroke and coma [36].

The initiation of therapeutic plasma exchange in the 1970s greatly transformed the outcome of the disease, reducing the fatal incidence to 5–16% [37]. iTTP patients have a better overall prognosis than other TMAs, including hospitalization time, quick platelet count recovery, and overall survival [38]. Nonetheless, disease exacerbations (platelet count drops, demanding PEX resumption within 30 days) and relapses (recurrence after 30 days) remain the most significant difficulties in the management of TTP [39].

## 2. Clinical Diagnosis

The diagnosis of TTP is based on clinical manifestations and laboratory results [40]. High suspicion of TTP is valid in patients with thrombocytopenia and microangiopathic hemolytic anemia, despite variable severity and presentation of ischemic organ damage (often brain and kidneys), and needs to be differentially diagnosed from other life-threatening TMAs and secondary TTP. Evidence of <10–20% ADAMTS13 activity is indicative and a pre-requisite of an acute TTP diagnosis [2], as it is a test with high sensitivity (97%) and specificity (100% from other TMAs) [38]. Moreover, low ADAMTS13 activity observed in TTP needs to be differentiated from aggressive cancer, sepsis, pregnancy, and liver disease [41]. Nonetheless, ADAMTS13 activity level testing is not available in all hospitals, and due to ELISA testing, results may require 2–5 days. Given that TTP is considered a medical emergency, waiting for ADAMTS13 activity results is not recommended, as therapeutic plasma exchange may be life-saving in a patient with high suspicion of TTP [20].

Major advances in TTP understanding have occurred over the last fifteen years. Several clinical scoring systems were developed in this context to aid in the rapid identification of patients suffering from severe ADAMTS13 deficiency. The French TMA and the PLASMIC score are the validated, predictive scores of TTP most frequently used [42]. These scores were not developed to substitute for ADAMTS13 activity testing, which is vital in the confirmation and follow-up of TTP, but to facilitate the clinical diagnosis of this life-threatening disease.

The French TMA score showed that the combination of creatinine levels of ≤200 µmol/L, platelet count ≤ 30 × 10^9^/L, and the presence of anti-nuclear antibodies has the most profound association with severe ADAMTS13 deficiency in patients younger than 55. The French score limitation is that it failed to associate also secondary TTP (HIV infection, cancer, transplantation); it focused solely on idiopathic TTP [43].

The Harvard TMA Research Collaborative developed the PLASMIC score to help diagnose and treat TTP. It is a 7-tier system used to categorize TMA patients according to their risk of severe ADAMTS13 deficiency, based on platelet count (<30 × 10^9^/L), hemolysis (indirect bilirubin >2 mg/dL or reticulocyte count >2.5% or undetectable haptoglobin), active cancer, previous transplantation, mean corpuscular volume (MCV) <90 fL, international normalized ration (INR) <1.5, and creatinine < 2.0 mg/dL [38,42]. A PLASMIC score of 0–4 indicates a low probability of TTP, whereas 6–7 is pathognomonic of a high probability of TTP with an approximately 62–82% risk of having severe ADAMTS13 deficiency [1]. The benefit for patients in developing countries from the sensitivity, specificity, and applicability of the PLASMIC score is undeniable, as it aids in early diagnosis and indication of patients who will most benefit from appropriate treatment [44,45]. The PLASMIC score can be used on patients with platelet count < 150 × 10^9^/L and the presence of schistocytes in the microscopic view of peripheral blood smear [42]. Moreover, recent cost-effective studies showed that the PLASMIC score application on suspected TTP patients during clinical decision-making reduced costs related to unregulated ADAMTS13 activity tests in several institutions [46,47]. 

A recent study showed that the sensitivity and specificity of these two clinical scores (the French score and the PLASMIC score) in the diagnosis of severe ADAMTS13 deficiency, and consequently of TTP, significantly decrease with age, as renal impairment is more profound in comorbid, older patients [48], thrombocytopenia is less pronounced than younger patients [49], and MCV tends to increase in older patients [50]. A recent study compared the performance of the modified French score and the PLASMIC score and found the PLASMIC score superior (c-statistic 0.93 vs. 0.88 for the French score, *p* = 0.0032) [51]. As far as clinical diagnosis of iTTP in older patients is concerned, atypical neurological manifestations (indicative of end-organ ischemic damage) delay the diagnosis of iTTP and consequent treatment, resulting in significant short-term and long-term mortality of these patients [49].

**Figure 1:**
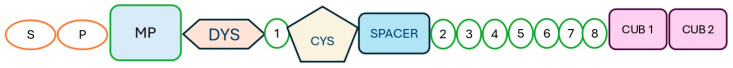
Schematic representation of the ADAMTS13 molecule structure. ADAMTS13 is a metalloprotease that consists of a signal peptide domain (S), a pro-peptide domain (P), a metalloprotease domain (MP), a disintegrin domain (DYS), a series of thrombospondin type 1 (TSP 1), a cysteine-rich domain (CYS), and a spacer domain (Spacer). Additionally, the C-tail consists of seven more repeats of thrombospondin type 1 (TSP 2–8) and CUB domains (CUB 1–2) [55,56].

Evaluation of ADAMTS13 qualities, such as activity, conformation status, antibodies, and antigen, is highly significant in the TTP diagnosis, as open conformation of ADAMTS13 is a subtle marker of TTP confirmation, in cases where ADAMTS13 activity is borderline low (i.e., 10–20%), or when levels of anti-ADAMTS13 inhibitors remain insignificant [11,52] (Figure 1). Regarding disease severity and outcome prognosis, ADAMTS13 qualities, as well as non-ADAMTS13 indices (troponin [12], endothelial activation with big endothelin-1 and syndecan-1 [53,54], and Glasgow coma scale [12]) have been proposed as prognostic risk factors for TTP outcome [11].

## 3. Management of TTP

### 3.1. Management of Acute TTP

Treatment options for acute iTTP include therapeutic plasma exchange and steroids, ideally started within 4–8 h from the diagnosis, as the disease has a high mortality risk. Evidence suggests that platelet transfusion in the acute TTP setting is associated with elevated mortality and, thus, should be avoided [57]. Therapeutic plasma exchange should be initiated at high suspicion of TTP and, usually, while waiting for confirmatory results of low ADAMTS13 activity. PEX theoretically removes anti-ADAMTS13 autoantibodies from the patient’s serum while replacing the deficient ADAMTS13 enzyme. Nonetheless, this approach does not address the pressing underlying autoimmune situation [20] that concurrent immunosuppression with corticosteroids does. Thus, the patient initially receives high doses of corticosteroids, followed by tapering over 20–30 days after an adequate clinical response (normalization of platelets and ADAMTS13 activity).

Caplacizumab is a monoclonal antibody that stops the von Willebrand factor (vWF)—platelet interaction by attachment to the vWF, resulting in normalization of the platelet count and prevention of microthombi formation [39,58]. It currently has a level of recommendation 1A in the acute setting [59], as it expedites platelet normalization [60], minimizes mortality in iTTP [61], and decreases exacerbations, disease refractoriness, hospitalization time [43,62,63], plasma transfusions, and the plasma volume used [64,65], compared to historical cohorts. The phase 2 study TITAN and phase 3 study HERCULES showed that caplacizumab reduces mortality and refractory disease when compared to placebo [66]. Caplacizumab reverses the patient’s clinical situation, but it does not change the immune disease process. Continuous ADAMTS13 severe deficiency—past 30 days of appropriate treatment—is associated with disease relapse, and caplacizumab may be continued [64] despite its significant cost (approximately $7700 per dose) [67]. A recent study showed that front-line treatment with caplacizumab, compared to delayed treatment with the agent, relates to lower costs due to shorter hospital stays and less usage of healthcare resources [68].

Rituximab is a human, anti-CD20, chimeric monoclonal antibody that suppresses the production of ADAMTS13 inhibitors by depletion of the B lymphocytes [69]. It currently has a level of recommendation 2B [59], and its use is saved for refractory or recurrent TTP [70]. Rituximab is given at a weekly dose of 375 mg/m^2^ over four weeks, based on lymphoma management guidelines, although further immunosuppression is guaranteed in failed ADAMTS13 activity level normalization [57]. Moreover, a continuous low dose (100 mg weekly) of rituximab has been shown to act effectively as adjuvant treatment in the acute TTP setting [71]. 

Alternatively, other humanized anti-CD20 antibodies, obinutuzumab and ofatumumab, are currently offered to patients with refractory or highly relapsing TTP who do not respond to or are intolerant to rituximab [72]. Ofatumumab is offered at three weekly doses of 300 mg, followed by 1 g and 1 g. It has also been given in the acute TTP setting to a patient with a previously reported allergic reaction to ritixumab [73]. Robertz et al. reported using obinutuzumab, a type-2 anti-CD20 antibody, at three weekly doses of 1000 mg in TTP patients who presented with serum sickness during preemptive treatment with rituximab [74].

Other TTP-directed treatments include daratumumab [75], an anti-CD38 antibody, and bortezomib [76], a protease inhibitor, in patients with continuous ADAMTS13 deficiency and persistent presence of anti-ADAMTS13 inhibitors despite adequate anti-CD20 inhibition.

A new drug, recombinant ADAMTS13 (rADAMTS13), has recently gained FDA approval for the prophylactic and on-demand management of congenital TTP [77,78] in adults and children, replacing the defective ADAMTS13 protein. The efficacy and safety profile were assessed in a recent phase 3, open-label, cross-over trial, which showed that no patients developed acute TTP or neutralizing antibodies when administered at 40 IU per kilo body weight, with raised ADAMTS13 activity (101%) after treatment with recombinant ADAMTS13, compared to standard treatment (19%) [78]. Another recent report supported using rADAMTS13 in iTTP patients as a cutting-edge adjunctive therapy. This report resulted in a prompt cease of the disease activity and quick recuperation in a severely affected patient who suffered from refractory iTTP [79]. Dadoun et al. recently used rADAMTS13 as a rescue therapy for acute cTTP in a pregnant patient with a successful outcome, paving the way for the use of this new drug in this distinct patient population [80].

Therapeutic PEX has improved the survival rates of acute TTP patients (from <15% to >80%) and is, therefore, considered the cornerstone of acute TTP treatment. It acts by removing the von Willebrand factor and ADAMTS13 autoantibodies while restoring the indispensable, functional ADAMTS13 levels. In the era without therapeutic plasma exchange for acquired TTP, the combination of caplacizumab, together with glucocorticoids and rituximab, may prove an effective treatment for TTP patients who are unwilling (due to religious convictions, e.g., Jehovah’s Witness patients) or unable to receive plasma exchange (lack of availability, inaccessible venous access, previous severe immune reaction to blood products) [81]. More trials are needed to, firstly, identify the subset of TTP patients who will be treated safely and effectively without PEX [82] and, secondly, to assess the application of caplacizumab, glucocorticoids, and rituximab in this selected TTP patient group [83]. 

Emerging therapies in TTP include a sleeping beauty transposon system for the long-term production of functional ADAMTS13 in preclinical mice models [84] and an aptamer to von Willebrand factor to minimize thrombosis [85]. These novel drugs may hold the key to unlocking the future of a cure for TTP [86].

Over recent years, the management of acute TTP has significantly improved, decreasing the mortality rates from 90% to 15%. Notwithstanding, the foregoing, unpredictably severe relapse in approximately half of the treated patients remains a cause of mortality or serious, long-term complications [87,88]. The relapse risk appears to be higher in cases with ADAMTS13 activity < 10–20% while in clinical remission.

Preemptive rituximab therapy is used in patients at high risk of TTP relapse, like patients after remission with persistently low ADAMTS13 activity [89], to minimize the relapse rate by up to 75% [90]. The number needed to treat to prevent one patient from relapsing is 3.3, whereas adding rituximab in initiating treatment minimizes the absolute relapse risk by 30% [91]. Its preemptive application in asymptomatic TTP survivors who are experiencing an ADAMTS13 level activity drop has presented as a promising strategy [92]. Preemptive rituximab administration during the management of TTP is, in general, well tolerated, with mild infusion reactions most frequently reported [93]. However, this novel approach presents several clinical dilemmas, including rituximab dosing, infusion schedule, and long-term follow-up regimens.

TTP survivors reporting more severe reactions to preemptive rituximab therapy or serum sickness should explore other anti-CD20 alternatives. Doyle et al. recently reported the preemptive use of obinutuzumab and ofatumumab to prevent a TTP relapse in 15 patients (26 episodes), achieving a sustainable remission in 15 days with minimal adverse events [72]. Last but not least, it is noteworthy that, however promising rADAMTS13 appears to be as a preemptive treatment in cTTP, there is not enough evidence to support its use in TTP patients in clinical remission at high risk of relapsing [78].

### 3.2. Follow-Up after Remission

The main goal of the intense follow-up of TTP survivors is the avoidance of clinical relapse (30–50%) and relevant ischemic end-organ damage, which is achieved by regular ADAMTS13 activity measurements, anti-ADAMTS13 antibody levels (in iTTP) monitoring, and blood count and hemolysis parameters assessment. Meticulous follow-up includes regular measurements of ADAMTS13 activity during the remission period. Some authors suggest measurements every 3 months for a maximum 12-month period [94], while others suggest longer-term follow-up [93,95]. Measurements’ intervals should be adjusted accordingly in patients at high risk of relapse (e.g., experiencing a sudden drop in ADAMTS13 activity) [93]. In case of detection of <10% ADAMTS13 activity, rituximab or other anti-CD20 treatment should be started to prevent a relapse [93]. Follow-up includes neurocognitive and psychology assessments to timely recognize anxiety and or depression, which is very common in these patients [96]. However, interventional studies assessing the TTP survivors’ group are currently missing from the literature, despite the availability of registry studies showing the neuropsychological burden of the disease, and this should comprise a part of future research.

**Figure 2:**
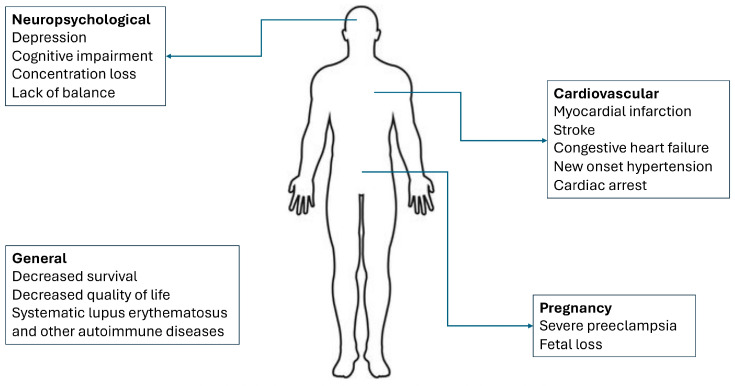
Adverse events in iTTP survivors.

TTP survivors exhibit more frequently chronic comorbidities (Figure 2), such as new onset hypertension [97], cardiovascular disease (acute myocardial infarction, congestive heart failure, arrhythmias) [98,99], neurocognitive deficits, concentration loss, lack of balance, and cognitive impairment [100]. Moreover, even without additional health issues, TTP survivors have lower scores in health-related quality of life questionnaires and increased anxiety and depression compared to the general population [101]. Premature death [97] is a significant health risk for this distinct patient group, as major cardiovascular events are the leading cause of death [101,102]. Lastly, severe preeclampsia and fetal loss are increased in TTP survivors’ subsequent pregnancies [103].

Patient follow-up is more than clinical visits and ADAMTS13 measurements. Historically, specialist nurses have played a crucial role in caring for TTP patients, mainly in facilitating treatment, coordinating care, promoting patient advocacy, encouraging continuous patient service improvement, and educating their wider clinical environment [104]. Moreover, patients’ advocates and support organizations aid in disseminating useful information and patients’ stories, raise money for research, and provide support to TTP sufferers and their families. A recent study explored the significance of including patients’ voices with hereditary TTP in their care, as they pointed to salient symptoms and their impact while expressing their need for safer and more efficient treatment options [105].

### 3.3. Relapse

Relapse is defined as a new TTP episode with thrombocytopenia, microangiopathic anemia, and low ADAMTS13 activity 30 days or more after treatment completion [106]. An established risk factor for disease relapse is persistent or recurrent deficient ADAMTS13 activity in iTTP acute phase survivors during follow-up [107,108]. Evidence shows that patients with continuously low ADAMTS13 activity and detection of ADAMTS13 inhibitors during the acute episode of TTP and the remission period present with earlier TTP relapse [11]. 

Research has shown that patients with ADAMTS13 activity of less than 70% during clinical remission are five times more likely to have a stroke [109]. Another study showed that all-cause mortality was associated with suboptimal ADAMTS13 activity levels but did not reach statistical significance, probably due to the small sample size of the study [110]. This highlights the need to achieve not only the patient’s clinical remission but also ADAMTS13 activity levels’ normalization. Interestingly, it also indicates the need for continuous follow-up of TTP survivors, in line with the International Society of Thrombosis and Hemostasis (ISTH) good practice guidelines, which recommend serial ADAMTS13 activity evaluation every 3–6 months [111]. This approach certainly detects early a possible change in ADAMTS13 activity and allows the initiation of available preemptive therapies to prevent a relapse [90]. Further studies will shed light on the optimal follow-up management to prevent relapse and reduce mortality and morbidity in TTP survivors.

## 4. Conclusions

In conclusion, developments in rare diseases, such as TTP, concerning diagnostics and therapeutics are associated with long-lasting challenges in this field. The focus in TTP has shifted from surviving the individual, acute episode to survivorship. As their survivorship increases, so will the pool of TTP survivors in the future. However, patients, during follow-up, are at increased risk of adverse events, ranging from stroke, cognitive impairment, and depression to poorer quality of life. Questions regarding optimal follow-up duration and relapse prevention strategies will be more pressing [112]. Regarding the treatment of TTP, patients may now receive novel and innovative treatments while accessing clinical research diversely and inclusively. Moreover, the personalized selection of patients who will most benefit from these specific treatments is pivotal in the era of precision medicine. Nonetheless, it is important to investigate the underlying mechanisms implicated in meager outcomes in TTP survivors in order to improve long-term care. Overcoming these challenges requires selective efforts from various active parties in the terrain of TTP, including patients’ advocates.

## Figures and Tables

**Table 1:** Method’s characteristics of measurement ADAMTS13 activity levels.

	ELISA	FRETS-VWF73	AcuSTAR	Ceveron FRET
Time to results	6 h	1.5 h	1 h	1 h
Requirements	ELISA plate readers [30]	specific analytical platforms	fully automated- needs specialized equipment	specific analytical platforms
Disadvantages	manual and time-consuming	reduced reaction rates in hyper-bilirubinermic plasmas [27]	Underestimates ADAMTS13 levels in the high assay range values (>40%) [31,32]	gold-standard
